# Milk yield prediction in Friesian cows using linear and flexible discriminant analysis under assumptions violations

**DOI:** 10.1186/s12917-024-04234-1

**Published:** 2024-09-06

**Authors:** Sherif A. Moawed, Esraa Mahrous, Ahmed Elaswad, Hagar F. Gouda, Ahmed Fathy

**Affiliations:** 1 Department of Animal Wealth Development, Biostatistics Division, Faculty of Veterinary Medicine, Suez Canal University, Ismailia, 41522 Egypt; 2 Center of Excellence in Marine Biotechnology, Sultan Qaboos University, Muscat 123, Oman; 3 Animal Wealth Development Department (Biostatistics Subdivision), Faculty of Veterinary Medicine, Zagazig University, Sharkia, 44511 Egypt

**Keywords:** Discriminant analysis, Flexible discriminant, Assumptions, Classification, Milk yield

## Abstract

**Background:**

The application of novel technologies is now widely used to assist in making optimal decisions. This study aimed to evaluate the performance of linear discriminant analysis (LDA) and flexible discriminant analysis (FDA) in classifying and predicting Friesian cattle’s milk production into low ($\:<$4500 kg), medium (4500–7500 kg), and high ($\:>$7500 kg) categories. A total of 3793 lactation records from cows calved between 2009 and 2020 were collected to examine some predictors such as age at first calving (AFC), lactation order (LO), days open (DO), days in milk (DIM), dry period (DP), calving season (CFS), 305-day milk yield (305-MY), calving interval (CI), and total breeding per conception (TBRD).

**Results:**

The comparison between LDA and FDA models was based on the significance of coefficients, total accuracy, sensitivity, precision, and F1-score. The LDA results revealed that DIM and 305-MY were the significant (*P* < 0.001) contributors for data classification, while the FDA was a lactation order. Classification accuracy results showed that the FDA model performed better than the LDA model in expressing accuracies of correctly classified cases as well as overall classification accuracy of milk yield. The FDA model outperformed LDA in both accuracy and F1-score. It achieved an accuracy of 82% compared to LDA’s 71%. Similarly, the F1-score improved from a range of 0.667 to 0.79 for LDA to a higher range of 0.81 to 0.83 for FDA.

**Conclusion:**

The findings of this study demonstrated that FDA was more resistant than LDA in case of assumption violations. Furthermore, the current study showed the feasibility and efficacy of LDA and FDA in interpreting and predicting livestock datasets.

## Background

Screening milk yield by investigating factors affecting production is critical for breeders. Large datasets, particularly for all animal parities, increase the challenge of prediction due to high dimensions. So, selecting the appropriate statistical method is crucial for analyzing biomedical data. Inappropriate methodological choices can lead to misleading interpretations and unsubstantiated conclusions. Understanding the assumptions and conditions of statistical methods is essential for selecting the appropriate method for data analysis. By doing this, researchers can ensure accurate and reliable data interpretation. Along with knowledge of statistical technologies, an additional important consideration is data type and nature, as well as the study’s goal [1]. In the case of categorical dependent variables, Discriminant analysis is primarily used to categorize data into two or more groups.

Discriminant analysis focuses on identifying significant factors for group distinction and categorizing unlabeled items. It involves two stages: separation and allocation. Separation aims to establish discriminant functions to separate groups, while allocation uses these functions to allocate unclassified items to recognized categories [2]. Additionally, it is carried out by computing the weights for each explanatory variable, which are calculated as between-group variance in relation to within-group variance [3]. Discriminant analysis is critical in determining the factors influencing milk production [4–6].

Various discriminant functions have been investigated over time, but they all serve the same purpose. Specific discriminant functions are required in certain circumstances. Linear discriminant analysis, Quadratic discriminant analysis, and Regularized discriminant analysis are examples of parametric discriminant analysis, while Flexible discriminant analysis and K-Nearest Neighbor analysis are examples of nonparametric discriminant analysis [7]. Linear methods are commonly used but may not always be sufficient in certain cases for accurate classification and error reduction. So, selecting an alternative approach can improve classification accuracy.

The flexible discriminant model (FDA) is a generalized linear discriminant analysis (LDA) method that can create non-linear boundaries for classes. The FDA’s inspiration originates from the connection between LDA, canonical correlation analysis, as well as optimal scoring. The primary difference is that the FDA can incorporate quadratic and bilinear predictors as well as nonlinear decision boundaries [8]. FDA is a valuable data prediction and classification method, and it is expected to draw the interest of medical investigators [9].

The current study aims to predict Friesian cow milk yield using linear discriminant analysis, use a more flexible classification model (flexible discriminant model) to accommodate the assumptions violation, and compare the performance of both models in anticipating and categorizing milk yield.

## Materials and methods

### Data collection and management

A total of 3793 dairy records were obtained for this study to investigate the significant variables influencing milk production in Friesian cows raised in Egypt. The data were gathered from one of the large farms in the El-Dakahlia governorate of Egypt. This investigation’s materials included records of dairy cows that calved between 2009 and 2020. Cows were mechanically milked three times per day under an automatic milking system. The daily milk yield was estimated using the sum of these three values. This study included cows that had completed at least one lactation. The lactation records and all other data are computer-based and updated regularly. On the farms, all cows were occasionally kept on unclean floors, in open yards, or slenderly covered yards with access to cool spraying during the summer. Animals were fed rations with a crude protein content of about 18%, according to National Research Council [10]. Isolation units were provided for sick and suspected animals and calving units were used for parturition. Storage units were also provided for feed and equipment. The calf-rearing system was an indoor system, allowing calves to stay with their dams and suckle for a limited period each day.

### Investigated traits

The research’s data were used to discover the major factors affecting milk yield in Friesians using linear and flexible discriminant models. As a result, the variables under consideration were actual total milk yield (kg), which was divided into three categories: low (< 4500 kg), medium (4500–7500 kg), and high (> 7500 kg), and considered the dependent variable of interest, while the rest variables considered as predictors include age at first calving (month), lactation order of eight parities (P1-P8), days open (days), dry period (days), days in milk (days), 305-day milk yield (kg), calving season (spring – summer – autumn- winter), calving interval (month), and number of breeding per conception (number). Furthermore, the categorization of milk yield variable and the selection of independent variables have been carried out based on previous authors’ recommendations [11–16].

### Data preprocessing

Data were initially checked for missing values as well as outliers. The data showed an imbalance in the number of the three levels of the outcome variable, which ranged between 3485 for the highly-producing cows, 287 for the medium class, and 21 for the low-producing category. The data was split randomly into two parts: 80% of the data was the training set for model creation and adjustment, and the second part was the testing set, representing 20% of the data for model validation and performance. The train set was processed by random over sampling (ROS) “up sampling” using the Caret package. The chi-square test and multinomial logistic regression were used to select variables for multivariate analysis, with all variables showing high significance levels (*p* < 0.001). For the LDA, the values of the different parameters were also centered and scaled to minimize data variability. Linear and flexible discriminant analysis models were used to classify cow milk yield into high, moderate, and low categories, creating a powerful model for future prediction.

### Linear discriminant analysis

Linear Discriminant Analysis is a parametric method used to identify the weights of independent variables that best distinguish between two or more mutually exclusive and exhaustive groups of cases. LDA is also a hard classification approach. Hard classifiers only define a hard classification boundary for each group based on the explanatory variables. A new item is assigned to a group based on characteristics that fit within the group’s characteristics [17]. Each group has a mean vector that is calculated using the attribute vectors of all the objects in the group. A test observation is assigned to the class whose centroid is the closest, where distance can be expressed as a Mahalanobis metric using the pooled within-group covariance matrix [18]. LDA functions are orthogonal to previous ones, and a number of discriminant functions of either G-1 or J, whichever is less, where G represents the categories number of the dependent variable and J represents the exploratory variables number [19]. The linear discriminant model is provided below:

1 $$\:{D}_{jk}={b}_{o}+\:{b}_{1}{x}_{1k}+{b}_{2}{x}_{2k}+\dots\:+{b}_{n}{x}_{ik}$$

Where: $\:{D}_{jk}$ is the discriminant score of discriminant function *j* for the milk yield level *k*, $\:{b}_{o}\:is\:$a constant term, $\:{b}_{i}$ is the $\:{i}^{th}$ predictor’s discriminant coefficient, and $\:{x}_{ik}\:$represent the $\:{i}^{th}$ explanatory variable (AFC, LO, DO, DIM, DP, CFS, 305-MY, CI, TBRD) for the milk yield level *k*.

The function generates discriminant scores, which estimate predicted probabilities for each categorical outcome variable. These scores, along with group means (centroids), aid in classifying cases into groups. A variable’s significance in explaining an outcome is indicated by large coefficients [20]. LDA has limitations since great accuracy can be achieved with strong multivariate normality assumptions and equal covariance matrices. However, these assumptions are uncommon in practice [2].

### Flexible discriminant analysis

Flexible discriminant analysis is a non-parametric generalization of linear discriminant analysis. It can be regarded as a technique of postprocessing a multi-outcome regression with an optimal scoring approach. The FDA procedure is similar to LDA, replacing linear regression with non-parametric or semi-parametric regression, allowing for various regression tools and discrimination rules. This enable a more flexible class boundaries and better discrimination [21, 22]. FDA was used to develop a coordinate system capable of distinguishing between all three levels of milk yield. To maximize separation between the data points of various milk yield types and to minimize variation among the data points within each milk production type, the data were mapped onto a two-dimensional plane. To determine the decision boundary, a regression is run, with the parameters set to reduce the average-squared residual (ASR) [22].

2 $$\begin{gathered} ASR = \:\frac{1}{N}\sum {\:_{k = 1}^K} \hfill \\\left[ {\sum {\:_{i = 1}^N} {{\left( {\theta {\:_k}\left( {{g_i}} \right) - \eta {\:_k}\left( {{x_i}} \right)\:} \right)}^2} + \lambda \:\:L\:\left( {\eta {\:_k}} \right)} \right] \hfill \\ \end{gathered}$$

Where the function θ allocates scores for the most accurate prediction of transformed class labels, N denotes the number of observations, K denotes the number of categories, η is the regression fit using nonparametric regression techniques, and L denotes the regularization penalty function used to penalize roughness in the regression fit, with a weighting determined by parameter λ. The FDA permits the use of nonparametric regression techniques, including multivariate additive splines (MARS) [23], neural networks [24], and generalized additive models [22] in model fitting. MARS is a spline regression that substitutes basis functions for the initial data as predictors. The MARS basis function change permits to specifically blank out regions of a variable by setting them to zero. This enables MARS to concentrate on specific sub-regions of the data, obtain optimal variables and interactions, and handle complex data structures with high dimensions [25].

In brief, the calculations are as follows:

1. Multivariate nonparametric regression: provide fitted values $\:\widehat{Y}$ for a multiresponse variable, adaptive nonparametric regression of Y on X.$\:{S}_{\:\lambda\:\:}$ is the linear operator that best fits the final model, and $\:{{\eta\:}^{*}}_{\mathcal{l}}\:$is the vector of fitted regression functions, shall be considered.
2. Optimal scores: calculate the eigen-decomposition of $\:{Y}^{T}\widehat{Y}={Y}^{T}{S}_{\:\lambda\:\:\:}Y$, in which the eigenvectors $\:{\Theta\:}$ are normalized, using the formula: $\:{{\Theta\:}}^{T}{D}_{\pi\:}{\Theta\:}\:=\:I.$ A diagonal matrix of the estimated class prior probabilities is calculated as $\:{D}_{\pi\:}=\:{Y}^{T}Y/N$
3. Update the statistical model from Step 1 by applying the optimal scores: $\:\eta\:\left(x\right)={{\Theta\:}}^{T}{\eta\:}^{*}\left(x\right).$

### Assumptions

The choice between LDA and FDA is more dependent on assumptions beyond each approach. In theory, the FDA is more flexible in terms of assumptions, especially variable distributions, and outliers. However, they both share some assumptions, such as observation independence and the absence of multicollinearity between predictors in datasets [26]. Hence, LDA required the testing of certain fundamental assumptions before its application. The independent variables were examined for multivariate normality, so, the predictors should be ratio or interval levels. Mahalanobis distance against chi-square was used to determine the presence of outliers. A simple scatter plot was used to assess the data’s linearity assumptions between the predictor and response variables [27]. The homogeneity of covariance matrices among dependent variable levels was verified using Box’s M test [28] while the variance inflation factor (VIF) was used to examine the multicollinearity of suggested discriminators; a VIF value of 1 indicates no correlation, a VIF value between 1 and 5 indicates moderate correlation, and a VIF value greater than 5 indicates strongly correlated [29].

### Classification model evaluation

According to Sokolova and Lapalme [30], the prediction model is evaluated using overall classification accuracy (ACC), which measures the ratio of correctly classified observations to total observations. Researchers often use other metrics like sensitivity (SEN) and precision (PREC) to measure the accuracy of classifiers. SEN is calculated as (TP / (TP + FN)), which is the percentage of target class labels correctly classified as desired [31, 32], while PREC is calculated as (TP / (TP + FP)), which is the proportion of correctly classified examples. TP represents true positives predicted by the classifier, TN represents true negatives, FP represents false positives, and FN represents false negatives [33, 34]. An additional metric is known as the F1-score, which is regarded as the harmonic mean of SEN and PREC. It examines the precision and sensitivity of the model in identifying positive cases (PREC) as well as its ability to prevent missing positive examples (SEN). The F1-score is defined as follows: $$\:\:\text{F}1-\text{s}\text{c}\text{o}\text{r}\text{e}=\left(\:2*SEN*PREC\right)/\left(SEN+PREC\right)$$ [30].

### Software and packages used

Linear discriminant analysis was performed using the MASS package while FDA using the MARS method was performed using the “fda” function from the “mda” package. All analyses were carried out using the R programming language [35] and SPSS version 25.

## Results

### Tests of assumptions

**Fig. 1:**
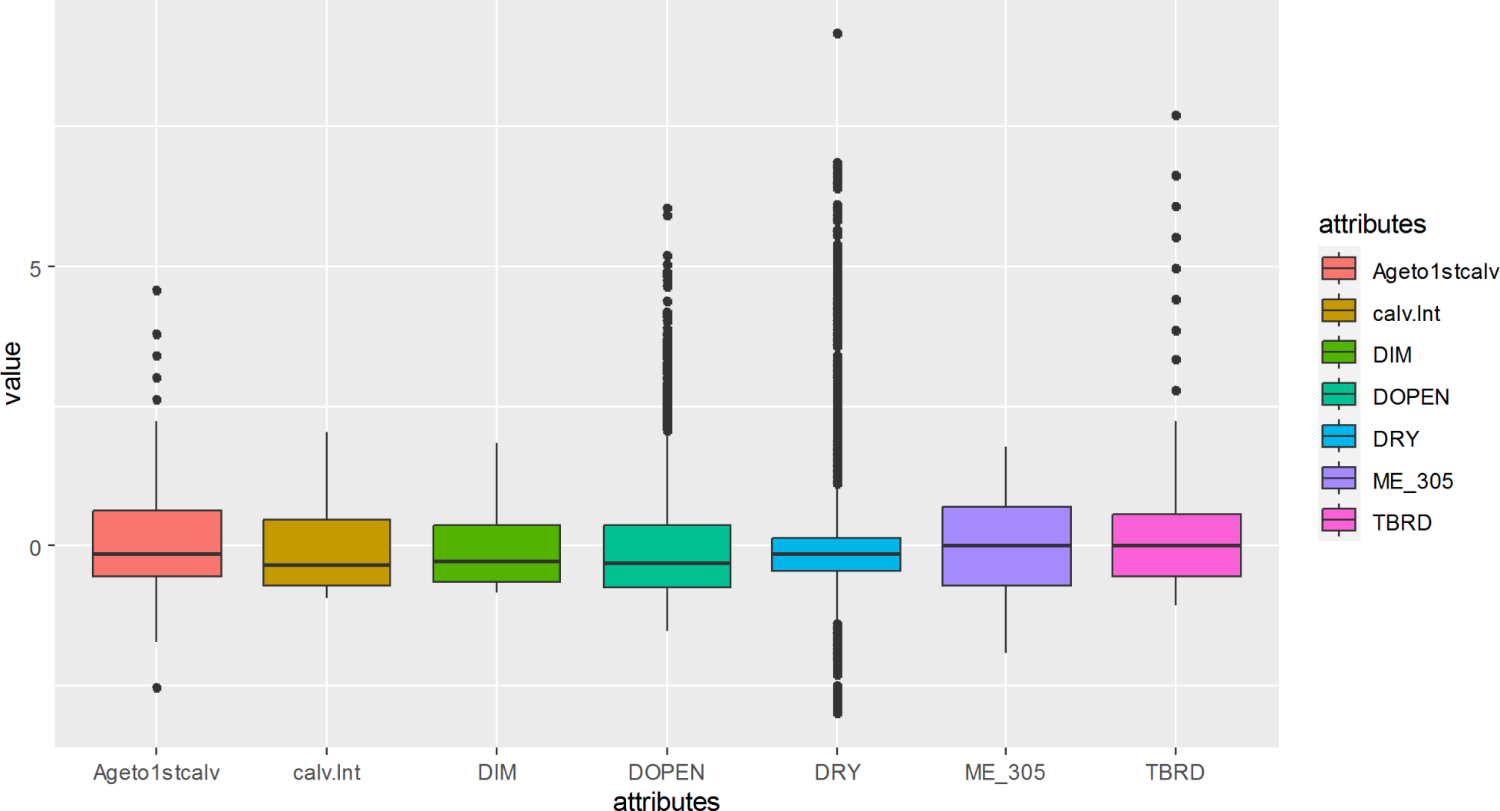
Box plot revealed that the data contains outliers in most of the predictors

**Fig. 2:**
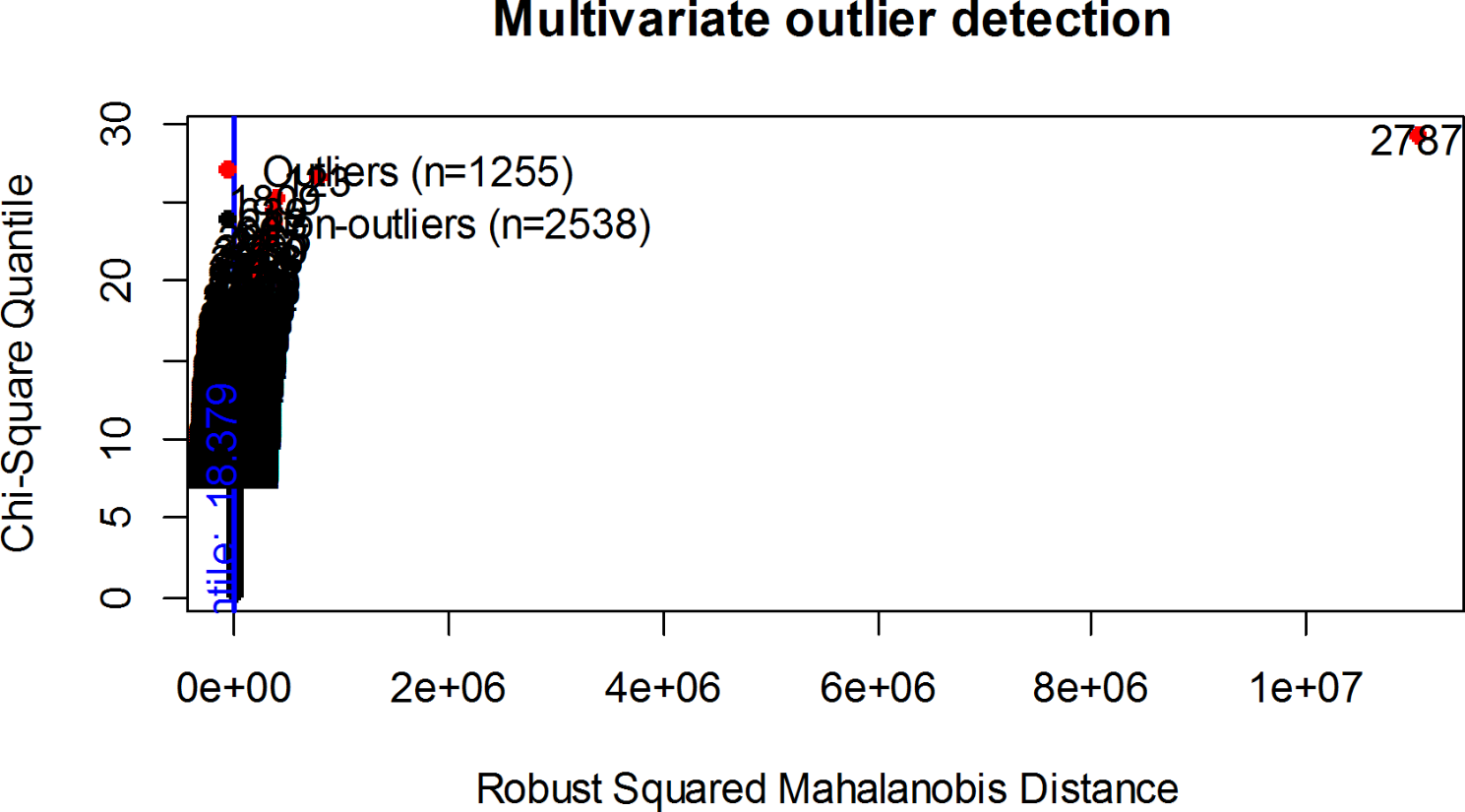
Multivariate outliers using Mahalanobis distance

First, the study checked the assumptions of LDA. There are no missing values in the data. Box plot indicated that the data contains univariate outliers in the majority of the explanatory variables as illustrated in Fig. 1. Whereas multivariate outlier based on Mahalanobis distance revealed that there are many outliers, as demonstrated in Fig. 2 below.

The homogeneity of covariance matrices was tested using the Box’s M statistic, which showed the violation of that assumption $\:({\text{B}\text{o}\text{x}}^{{\prime\:}}\text{s}\:\text{M}=342,\:\text{a}\text{n}\text{d}\:\text{p}<0.001\:).\:\:$Results indicated that the univariate normality of independent variables was violated while multivariate normality was met by the Henze-Zirkler test $\:(\text{H}\text{e}\text{n}\text{z}\text{e}-\text{Z}\text{i}\text{r}\text{k}\text{l}\text{e}\text{r}\:\:\text{v}\text{a}\text{l}\text{u}\text{e}=\:106.95,\:\text{p}\:<\:0.001)$. Nonetheless, the analysis was carried out in violation of this assumption as the discriminant analysis exhibits good results in face and object recognition, even though the normality assumption is frequently violated [36]. Scatter plots tested the linearity assumption, showing a linear relationship between the outcome variable and only a few independent variables. The VIF test was used, and there is a moderate degree of multicollinearity as $\:\text{V}\text{I}\text{F}\:\text{b}\text{e}\text{t}\text{w}\text{e}\text{e}\text{n}\:2\:\text{t}\text{o}\:5\:$ for three parameters (DO= 5.84, DIM= 3.95, and CI=2.36). As VIF does not indicate which pair of predictors are correlated, a correlation analysis was performed to determine which parameters are correlated. The results revealed that days open have a significant relationship with days in milk ($\:r=0.85$), and days open are also highly correlated with calving interval ($\:r=0.76$). As a result, we dropped the DO variable to eliminate multicollinearity.

### Linear discriminant analysis

The LDA model calculates group means and the probability of belonging to the different categories for each individual. In other words, the average of each explanatory variable within each category. These values could suggest that the DIM may have a greater influence on high milk production (61.6) than on medium (23.6) and low (37.5) milk production, as was also seen with 305-MY. The model’s group means indicate good separation ability between milk production levels (high, medium, and low), as displayed in Table (1).

**Table 1:** Group means for each predictor within each class using LDA

	P1	P2	P3	P4	P5	P6	P7	P8	AFC
Low	-0.124	0.288	-0.052	-0.032	-0.157	-0.105	-0.058	-0.024	-0.037
Medium	0.349	-0.320	-0.07117	-0.104	0.049	0.033	0.023	-0.024	-0.031
High	-0.208	0.010	0.144	0.124	0.106	0.061	0.028	0.035	0.061
	**Autumn**	**Spring**	**Summer**	**Winter**	**DIM**	**DRY**	**TBRD**	**CI**	**305-MY**
Low	0.108	-0.131	0.073	-0.092	-0.375	0.287	-0.025	-0.040	-0.752
Medium	-0.164	0.130	-0.014	0.093	-0.236	-0.062	-0.104	-0.196	-0.186
High	0.042	-0.014	-0.033	0.001	0.616	-0.251	0.127	0.245	0.949

The study found that all independent variables significantly contribute to data classification, indicating a significant difference between milk production levels. The Wilks’ lambda value decreases as the independent variable becomes more significant in the discrimination process. Therefore, as shown in Table (2), the most significant variables were 305-day milk yield ($\:\gamma\:$= 0.610), Days in milk ($\:\gamma\:$ = 0.909), and second parity ($\:\gamma\:$ = 0.930).

**Table 2:** Predictors’ role in explaining outcome using linear discriminant analysis

Predictors	Wilks’ Lambda	F	*P* value
P1	0.942	257.397	<0.001**
P2	0.930	314.533	<0.001**
P3	0.992	34.749	<0.001**
P4	0.992	32.764	<0.001**
P5	0.987	53.579	<0.001**
P6	0.994	25.014	<0.001**
P7	0.998	8.453	<0.001**
P8	0.999	3.007	<0.049*
Autumn	0.987	57.094	<0.001**
Spring	0.990	43.605	<0.001**
Summer	0.996	15.930	<0.001**
Winter	0.991	36.014	<0.001**
Days in milk	0.909	418.885	<0.001**
Dry period	0.937	282.220	<0.001**
Total number of breedings	0.975	105.043	<0.001**
Calving interval	0.957	186.571	<0.001**
305-MY	0.610	2670.33	<0.001**
Age at first calving	0.996	17.919	<0.001**

**Coefficient is significant at a 0.001 level of significance *(P < 0.001)*

*Coefficient is significant at a 0.05 level of significance *(P < 0.05)*

Table (3) displays the model’s coefficients, which are linear combinations of predictor variables used to create the LDA decision rule. Standardized canonical discriminant coefficient was used to rank the significance of each variable. A high standardized discriminant function coefficient indicates significant differences between groups on a particular variable, so the first three parities are considered the most significant factors based on these coefficients. This result contradicts Wilk’s test results for the significance of predictors observed in Table (2), which show the impact of outliers and collinearity on the data. Therefore, we focused on the structure matrix as its results matched Wilk’s test’s rank of predictor importance. According to the factor loadings, 305-MY and DIM are important parameters of discrimination on $\:{\text{L}\text{D}}_{1}$, whereas the first and second parities are on $\:{\text{L}\text{D}}_{2}$.

**Table 3:** Standardized and unstandardized discriminant coefficients for the predictors

Predictors	Structure (factor) coefficients			Standardized canonical discriminant function coefficients		Unstandardized canonical discriminant function coefficients	
	Function			Function		Function	
	I	II	Rank	I	II	I	II
P1	0.082	0.605	7	1.229	1.042	2.542	2.155
P2	0.089	-0.670	6	1.087	0.130	2.610	0.311
P3	-0.090	-0.098	16	0.667	0.357	2.129	1.138
P4	-0.092	-0.069	3	0.297	0.263	1.278	1.136
P5	-0.113	0.115	14	0.186	0.367	1.257	2.479
P6	-0.072	0.103	15	0.107	0.296	1.004	2.787
P7	-0.030	0.090	17	0.085	0. 213	1.386	3.458
P8 (base category)	-0.027	-0.024	5				
Spring	-0.038	0.246	11	-0.068	0.157	-0.198	0.457
Summer	0.057	-0.084	18	0.102	-0.172	0.222	-0.375
Autumn	0.003	-0.299	9	0.068	-0.375	0.147	-0.805
Winter (base category)	-0.034	0.224	12				
DIM	-0.344	-0.072	2	-0.234	0.106	-0.002	0.001
DP	0.258	-0.273	10	0.093	-0.204	0.003	-0.006
TBRD	-0.150	-0.200	13	0.003	-0.060	0.002	-0.043
CI	-0.173	-0.356	8	-0.069	-0.534	-0.027	-0.210
305-MY	-0.871	-0.066	1	-0.917	-0.036	0.000	0.000
AFC	-0.071	-0.001	4	0.005	-0.027	0.002	-0.012
Constant						1.207	2.218

*(P1-P8)* = Parity, *(Spring-Summer-Autumn-Winter)* = Season, *DIM* = Days in milk, *DP* = Dry period, *TBRD* = Total breedings per conception, *CI* = Calving interval, *305-MY* = 305-day milk yield, *AFC* = Age at first calving

Reference categories: Parity (P8), Season (Winter)

The canonical discriminant function is built using unstandardized coefficients:

$$\eqalign{& \>{\rm{L}}{{\rm{D}}_1} = \>\>1.21 + \>2.542{\rm{*}}\>{\rm{parity}}1\> + \>2.610{\rm{*parity}}2\> \cr & + \>2.129{\rm{*parity}}3\> + \>1.278{\rm{*parity}}4 + \>1.257{\rm{*parity}}5 \cr & + \>1.004{\rm{*parity}}6 + \>1.386{\rm{*parity}}7 + \>0.147{\rm{*Autumn}}\> \cr & - \>0.198{\rm{*Spring}}\> + \>0.222{\rm{*Summer}} - \>0.002{\rm{*DIM}} \cr & + \>0.003{\rm{*DP}}\> + \>0.002{\rm{*TBRD}}\> - \>0.027{\rm{*CI}}\> \cr & + \>0.00{\rm{*}}305{\rm{MY}} + 0.002{\rm{*AFC}}.\> \cr}$$

Similarly,

$$\eqalign{& \>{\rm{L}}{{\rm{D}}_2} = \>\>2.218 + \>2.155{\rm{*}}\>{\rm{parity}}1\> + \>0.311{\rm{*parity}}2\> \cr & + \>1.138{\rm{*parity}}3\> + \>1.136{\rm{*parity}}4 + \>2.479{\rm{*parity}}5 \cr & + \>2.787{\rm{*parity}}6 + \>3.458{\rm{*parity}}7 - \>0.805{\rm{*Autumn}}\> \cr & + \>0.457{\rm{*Spring}}\> - \>0.375{\rm{*Summer}} + \>0.001{\rm{*DIM}} \cr & - \>0.006{\rm{*DP}}\> - \>0.043{\rm{*TBRD}}\> - \>0.210{\rm{*CI}}\> \cr & + \>0.00{\rm{*}}305{\rm{MY}} - 0.012{\rm{*AFC}}. \cr}$$

There were two functions created: the first had a higher eigenvalue of 0.841 and explained 84.6% of the total variance in the outcome (milk yield level), and the second had an eigenvalue of 0.153 and explained 15.4% of the total variance in the outcome variable (Table 4). Eigenvalue is a measure of the variance that a function can explain. $\:{R}^{2}$ (Coefficient of multiple determination) is defined as the square of the canonical correlation value.

**Table 4:** Eigen values and Wilks’ Lambda for linear discriminant functions

Function	Eigenvalue	Percentage of Variance	Canonical Correlation	Wilks’ Lambda	Chi-square
I	0.841	84.6	0.676	0.471*	6287.215*
II	0.153	15.4	0.364	0.867*	1187.554*

The first discriminant function had mean scores for low milk production of 0.997, medium and high levels of 0.216 and − 1.217, respectively, while the second discriminant function had mean scores of -0.352 for low milk production and 0.546 and − 0.192 for medium and high levels (Table 5).

**Table 5:** Group centroids of low, medium and high-level milk yield by LDA

Group Centroids	Function I	Function II
Low	0.997	−0.352
Medium	0.216	0.546
High	-1.217	−0.192

The LDA model had a good discriminating accuracy of 71% in both the training and testing sets, which reveals that 71% of the milk production was properly classified by the model as displayed in Table (6). By 79%, 63%, and 71%, respectively, the model classifies high, medium, and low cases properly. Only 78.38% of all high cases have been identified by the model, 72.52% of all medium cases were detected by the model, and only 62.58% of all low cases were identified.

**Table 6:** LDA evaluation metrics for the model performance on train and test sets

Metrics	Train set			Test set (imbalanced data)			Test set (balanced data)		
	High	Medium	Low	High	Medium	Low	High	Medium	Low
ACC (%)	71.2			76			71.09		
SEN	0.79	0.64	0.72	0.78	0.61	0.00	0.79	0.64	0.71
PREC	0.78	0.73	0.63	0.987	0.28	0.00	0.78	0.73	0.63
F1-score	0.79	0.68	0.67	0.87	0.38	0.00	0.79	0.68	0.67

*ACC* Overall accuracy, *SEN* Sensitivity, *PREC* Precision

**Fig. 3:**
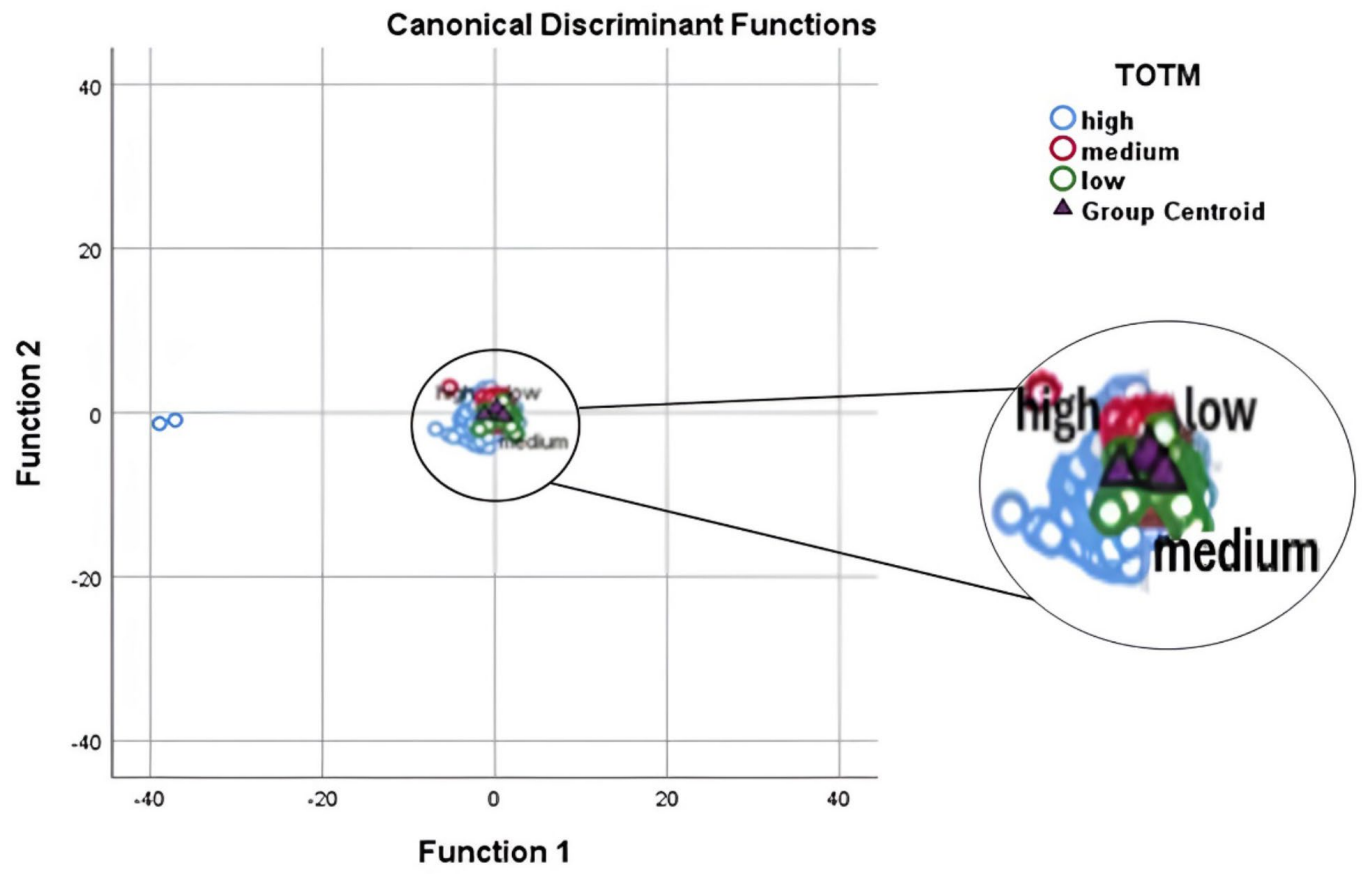
Linear discriminant analysis biplot revealed that the discrimination between group means isn’t good as group means are so close

The discriminant function plot revealed that the low level had the highest value on function I (Fig. 3). Function II played a minor role in the discrimination process compared to function I. Three milk yield levels membership appeared on the graph at nearly the same level, indicating poor discrimination between groups.

### Flexible discriminant analysis

Linear discriminant analysis requires a lot of assumptions to be held. As the results showed a violation of all assumptions, we applied FDA which tolerated the non-linearity of the data. Regarding multivariate normality, this assumption is rarely met. FDA using the MARS method was used, and the model discriminated the data by two dimensions with a misclassification error of 0.17. The first dimension accounted for 67.4% of the between-group variance, and by the second dimension totally reached 100%.

Table 7 displays the flexible discriminant analysis coefficients. The results revealed that the first and second parities are important parameters of discrimination on $\:{\text{F}\text{D}}_{1}$, whereas the sixth and seventh parities are on $\:{\text{F}\text{D}}_{2}$.

**Table 7:** Coefficients of the model for the first two dimensions of flexible discriminant analysis

Predictors	Unstandardized canonical discriminant function coefficients	
	Function	
	I	II
P1	-1.044	0.841
P2	-1.200	0.250
P3	-0.899	0.468
P4	-0.493	0.408
P5	-0.413	0.863
P6	-0.263	0.935
P7	-0.403	1.181
P8	0.553	-0.089
Spring	-0.118	-0.257
Summer	0.124	0.136
Autumn	NA	NA
Winter	-0.007	-0.005
DIM	NA	NA
DP	0.149	-0.167
TBRD	0.071	0.079
CI	-0.088	-0.029
305-MY	-0.026	0.004
AFC	-0.072	-0.104
DO	-0.127	-0.110
Constant	1.069	-0.532

*(P1-P8)* = Parity, *(Spring-Summer-Autumn-Winter)* = Season, *DIM* = Days in milk, *DP* = Dry period,

*TBRD* = Total breedings per conception, *CI* = Calving interval, *305-MY* = 305-day milk yield, *AFC* = Age

at first calving, *DO* = Days open

The canonical discriminant function is built using unstandardized coefficients:

$$\eqalign{& \>F{D_1} = \>\>1.069 - 1.044*\>parity1 - 1.200*parity2 \cr & - 0.899*parity3 - 0.493*parity4 - 0.413*parity5 \cr & - 0.263*parity6 - 0.403*parity7 + 0.553*parity8\> \cr & - 0.118*Spring\> + \>0.124*Summer - 0.007*Winter \cr & + \>0.149*DP\> + \>0.071*TBRD\> - 0.088*CI \cr & - 0.026*305MY - 0.072*AFC - 0.127*DO. \cr}$$

Similarly,

$$\eqalign{& \>F{D_2} = \>\> - 0.532 + \>0.841*\>parity1\> + \>0.250*parity2 \cr & + \>0.468*parity3\> + \>0.408*parity4 + \>0.863*parity5 \cr & + \>0.935*parity6 + \>1.181*parity7 - 0.089*parity8 \cr & - 0.257*Spring\> - \>0.136*Summer - 0.005*Winter \cr & - 0.167*DP\> - \>0.079*TBRD\> - 0.029*CI\> \cr & + \>0.004*305MY - 0.104*AFC - 0.110*DO. \cr}$$

**Fig. 4:**
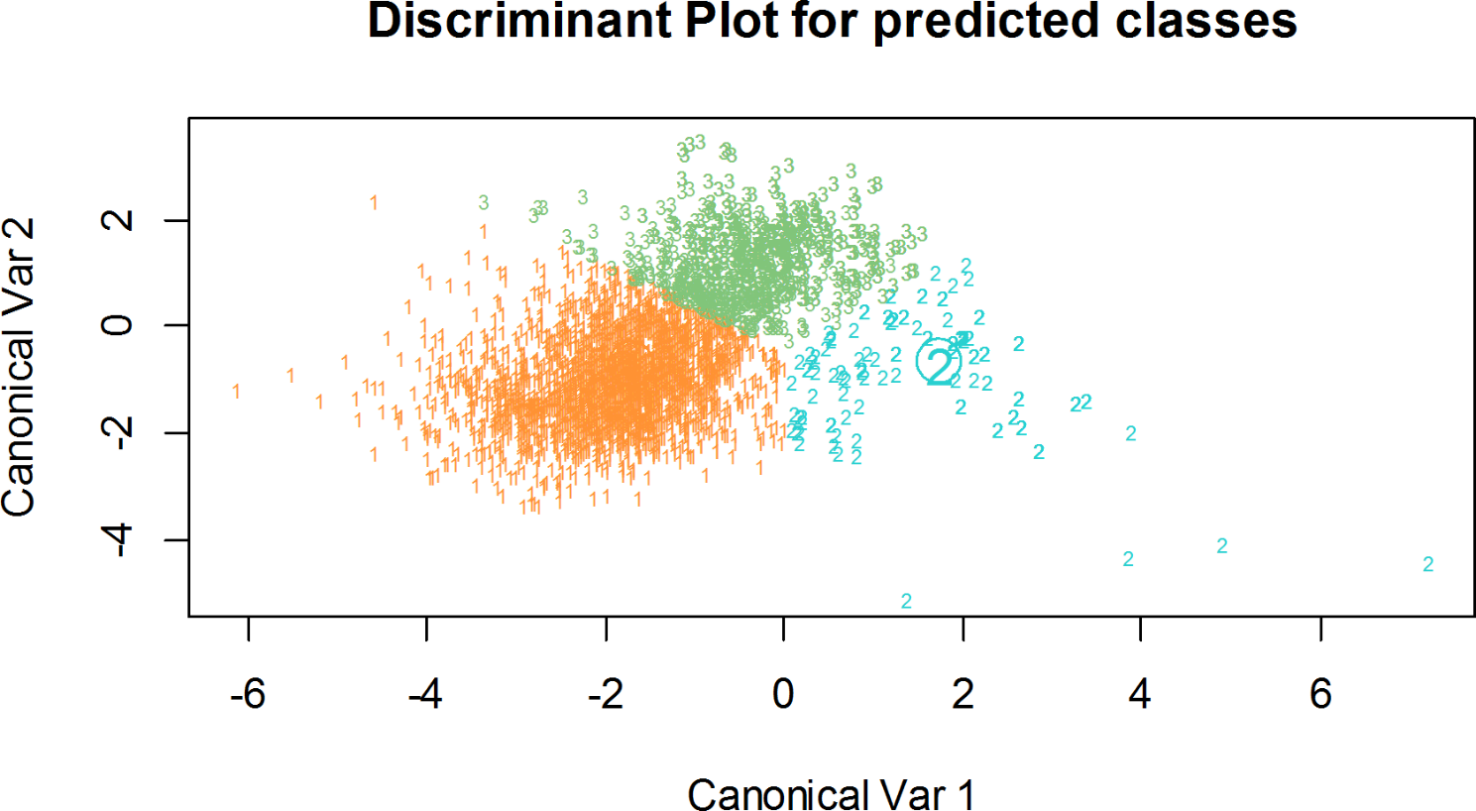
Flexible discriminant analysis’s discrimination biplot. The plot showed remarkable differentiation between the three categories of milk production. (1) The color orange stands for a high milk production. (2) The color blue stands for a medium category. (3) The color green stands for a low category

The class score means were presented in Table (8), with clear separation in the first dimension, however in the second dimension, the high and low categories are quite near. Overall, the separation appears to be satisfactory as seen in Fig (4).

**Table 8:** Group centroids of low, medium and high-level milk yield by FDA

Group Centroids	Function I	Function II
Low	1.748	-0.658
Medium	-0.055	1.386
High	-1.699	-0.725

The FDA model showed a superior discriminating accuracy of 82.45% and 83.19% in the training and testing sets, respectively, indicating accurate identification of milk production, as shown in Table (9). In the training set, the model correctly classifies high, medium, and low instances by 82.71%, 87.16%, and 77.39%, respectively. The model identified only 83.43% of all high cases, 75.90% of all medium cases, and 90.20% of all low cases.

**Table 9:** FDA evaluation metrics for the model performance on train and test sets

Metrics	Train set			Test set (imbalanced natural data)			Test set (balanced data)		
	High	Medium	Low	High	Medium	Low	High	Medium	Low
ACC (%)	82.45			83.60			83.19		
SEN	0.83	0.87	0.77	0.85	0.67	0.67	0.85	0.87	0.776
PREC	0.83	0.76	0.90	0.98	0.30	0.08	0.83	0.766	0.916
F1-score	0.83	0.81	0.83	0.91	0.41	0.14	0.84	0.816	0.84

*ACC* Overall accuracy, *SEN* Sensitivity, *PREC* Precision

### Comparison of classification techniques

The suggested LDA and FDA model’s robustness is validated through classification performance measures, each metric is reported for the three classes (high, moderate, and low). Notably, the FDA model achieved higher sensitivity (0.83, 0.87, and 0.77), precision (0.83, 0.759, and 0.90), and F1 score (0.83, 0.81, and 0.83) compared to LDA achieved the highest metrics for the (high) class (0.79, 0.78, and 0.79) for sensitivity, precision, and F1 score, respectively. Additionally, the FDA model exhibited a higher overall classification accuracy of 82% compared to LDA’s 71% (Tables 6 and 9). The model’s predictive power is low, and to improve results, the days open variable was removed. The findings, however, demonstrated that the assumptions were invalid. As a result, flexible discriminant analysis is created.

The evaluation on the test set revealed a clear advantage for the FDA model in terms of accuracy (82.45 − 83.6%) compared to LDA (71.09 − 76.0%). This difference suggests that LDA may be overfitting the training data, leading to lower generalizability on unseen data. While LDA achieved reasonable accuracy for the “high” class, its performance suffered for the “moderate” and “low” classes. This suggests an inability to accurately predict these categories. Conversely, the FDA model demonstrated good to high sensitivity across all classes (0.85, 0.67, and 0.67 for high, moderate, and low, respectively). While precision and F1-score for FDA decreased for moderate and low classes compared to the high class, they remained superior to LDA’s performance for all classes.

Our study aimed to evaluate the impact of assumption violations on model performance. However, we observed that both models, particularly LDA, struggled to predict some classes due to the inherent class imbalance in the data. To isolate the effect of assumption violation and ensure a fair comparison, we re-evaluated the models using a balanced test set. This step aimed to mitigate any potential confounding effect of class imbalance on our results and ensure our choice of model was based solely on the impact of assumption violations. The re-evaluated results, considering the balanced test set, further confirmed the superiority of the FDA model.

## Discussion

The current study classified and predicted Friesian milk yield using two classification methods, LDA and FDA. Based on the literature review, no similar studies were observed that implement and investigate LDA assumptions violations and compares performance with the FDA in prediction and classification. Based on past studies, the accurate classification method can vary depending on the data structure. This study highlights the importance of considering data characteristics and model assumptions when selecting classification methods for biological data analysis. El-Bayomi et al. [37] emphasize the value of choosing appropriate statistical techniques to ensure reliable and accurate outcomes.

The data initially displayed a severe imbalance, addressed through up-sampling, as recommended by Hassan et al. [38]. We opted against Synthetic Minority Over-sampling Technique (SMOTE) due to its potential drawbacks for sparse minority classes [39] and using SMOTE can disrupt the class separation that discriminant analysis relies on [40].

The LDA model exhibits violation of all assumptions and data contains outliers, even though the multivariate normality assumption is rarely met but still all other assumptions are violated. Therefore, we used another adaptable model that doesn’t necessitate linearity, and the results showed that FDA was superior to LDA in prediction and discrimination, which is supported by Moisen and Frescino [41], who noticed that the MARS model performed more accurately for predicting forest aspects than linear models.

If the data set is large and assumptions are violated, the FDA outperforms the LDA. Multidimensional data with outliers and multicollinearity can make it challenging for the LDA model performance to be applied. Application of machine learning algorithms such as artificial neural networks, random forests, and other machine learning models can improve results as stated by Gouda et al. [42], who reported that machine learning can handle both multidimensional and small datasets.

Selecting appropriate evaluation metrics is crucial and challenging, especially for imbalanced data. Traditional metrics like accuracy can be misleading in such scenarios Sokolova and Lapalme [30]. A model might achieve high accuracy by simply predicting the majority class most of the time, but this wouldn’t reflect its ability to correctly classify the minority class. Additionally, the Kappa value is inappropriate evaluation metric due to its potential bias and inaccuracy, as highlighted by previous study [43]. To address this challenge, we employed precision, recall, and F1 score as primary evaluation metrics. These metrics are well-suited for imbalanced datasets providing a more comprehensive picture of model performance [44].

Previous studies assessed LDA and FDA’s effectiveness using various criteria. As stated by Solberg [18], the FDA is a less computationally intensive algorithm for non-Gaussian feature classification without advanced parameter specifications. Öztürk and Özdamar [9] stated that FDA performed better than LDA. Furthermore, they concluded that FDA methods can be applied to nonnormal and heterogeneous data sets, while LDA should be applied to normal and homogeneous data sets. LDA is effective for classification but not for problems with non-normal distributions, according to Fukunaga [45]. Naghibi et al. [46] found that LDA is sensitive to outliers and cannot ensure a linear combination between the explained variable and other explanatory variables. So, the FDA exhibited the best performance among discriminant analysis models. However, more guidelines are required to choose between these methods properly.

According to LDA, the most important predictors in the discrimination process are DIM, 305-MY. This result is in agreement with the outcomes reported by Vijayakumar et al. [47] and Akkuş et al. [12] who stated that the amount of milk produced by Holstein cows was affected by lactation length. Some studies reported a negative correlation between DIM and milk yield [48, 49], others Mellado et al. [50] stated that there is low correlation between the total milk production and the 305-MY.While parities are a major factor in the FDA’s discriminating process, this result is consistent with Munim et al. [51], M’hamdi et al. [52], and Moawed and Abd El-Aziz [16] who observed a significant $\:(P<0.05)$ influence of parity on milk output. Nevertheless, the findings contrasted with those of Habib et al. [53], who indicated that lactation number did not affect milk output $\:(P>0.05)$.

## Conclusion

Linear discriminant analysis and flexible discriminant analysis are both suitable for classifying dairy cow data and yielding useful information, but their assumptions and methodology differ. Linear discriminant analysis requires more assumptions about underlying data, while the FDA doesn’t. Flexible discriminant analysis is more robust in cases of assumption violations. Overall, the FDA is a more powerful and adaptable classification method than LDA. However, the computing cost is higher, and the findings may be more challenging to understand.

## Data Availability

No datasets were generated or analysed during the current study.

## Abbreviations

LDA: Linear discriminant analysis
FDA: Flexible discriminant analysis
AFC: Age at first calving
LO: Lactation order
DO: Days open
DIM: Days in milk
DP: Dry period
CFS: Calving season
305-MY: 305-day milk yield
CI: Calving interval
TBRD: Total breedings per conception
MARS: Multivariate additive splines
VIF: Variance inflation factor
ACC: Overall classification accuracy
SEN: Sensitivity
PREC: Precision
SMOTE: Synthetic Minority Over-sampling Technique
ROS: Random over sampling
